# Chronic Immune Sensory Polyradiculoneuropathy-Plus With Diffuse Enlarged Dorsal Root Ganglion and Positive Serum Human Leukocyte Antigen (HLA)-B27

**DOI:** 10.7759/cureus.18320

**Published:** 2021-09-27

**Authors:** I-Chen Huang, Wei-Yu Chen, Jia-Ying Sung, Chih-Shan Huang

**Affiliations:** 1 Department of Neurology, Taipei Municipal Wan Fang Hospital, Taipei Medical University, Taipei, TWN; 2 Taipei Neuroscience Institute, Taipei Medical University, Taipei, TWN; 3 Department of Pathology, Taipei Municipal Wan Fang Hospital, Taipei Medical University, Taipei, TWN; 4 Department of Pathology, School of Medicine, College of Medicine, Taipei Medical University, Taipei, TWN; 5 Department of Neurology, School of Medicine, College of Medicine, Taipei Medical University, Taipei, TWN

**Keywords:** sural nerve biopsy, steroid treatment, chronic immune sensory polyradiculoneuropathy, azathioprine treatment, ataxia

## Abstract

The peripheral nerve is usually spared in chronic immune sensory polyradiculoneuropathy (CISP) according to a literature review; however, an extended-spectrum of CISP, CISP-plus, was introduced recently. Here we report a 29-year-old Taiwanese man who presented with numbness and hypoesthesia in all distal extremities, tightness sensation in the left posterior thigh, and sensory ataxia for three months. The clinical and neurophysiological examinations revealed proximal sensory abnormalities along with sural nerve involvement. The elevated protein level of cerebrospinal fluid (CSF) was noted and enlarged dorsal root ganglia were seen on the magnetic resonance imaging (MRI) of the whole spine. Autoimmune workup showed only positive human leukocyte antigen (HLA)-B27. Biopsy of the sural nerve revealed inflammatory demyelinating neuropathy. Only mild improvement was noted after methylprednisolone pulse therapy (1,000mg/day) for three days, and he was then treated with intravenous immunoglobulin with the dosage of 2g/kg-BW followed by azathioprine, and objective improvements were reported. Different from the previous case reports, CISP may also associate with peripheral nerve involvements. A sural nerve biopsy could assist the diagnosis. Further investigation is needed for the possible immune association between CISP-plus and HLA-B27.

## Introduction

Sensory ataxia theoretically may be due to the involvement of dorsal columns, dorsal root entry zone, dorsal root, dorsal root ganglia (DRG), and sensory nerves [1]. Chronic immune sensory polyradiculoneuropathy (CISP), also termed an ataxic form of chronic inflammatory demyelinating polyradiculoneuropathy (CIDP) in other reports [2], is a variant of CIDP, which can present with sensory ataxia due to affected dorsal roots proximal to the DRG [1]. According to the prior literature review, the peripheral nerve is usually spared in CISP; however, an extended-spectrum “CISP-plus” was introduced recently [3]. CISP-plus was identified as non-pure, sensory predominant, inflammatory polyradiculopathy and they are similar to CISP [3]. Some human leukocyte antigens (HLAs) expressed in CIDP patients were found, nevertheless, HLA-B27, a marker often associated with ankylosing spondylosis, was not reported in CIDP patients before [4]. Here we report a case of CISP-plus with sural nerve involvement confirmed by a sural nerve biopsy and positive HLA-B27.

## Case presentation

We reported a 29-year-old Taiwanese man who presented to the neurology clinic with numbness and hypoesthesia in all distal extremities, tightness sensation in the left posterior thigh, and sensory ataxia for three months. No history of alcohol consumption, any medication use, preceding infection, toxin exposure, or vaccination was retrieved. The neurological examination revealed normal muscle strength bilaterally. All four extremities were hyporeflexic. No cerebellar signs were present. The sensory examinations showed hypoesthesia by vibration, joint position, and pinprick tests at the bilateral palms and soles. The Romberg test was positive. The nerve conduction study (NCS) of the upper and lower extremities showed absent sensory nerve action potential (SNAP) waveforms at bilateral sural nerves (Table 1).

**Table 1:** The results of the pre- and post-treatment nerve conduction studies. The results of the pre-treatment nerve conduction studies of this patient showed absent SNAP in bilateral sural nerves and absent H reflex in bilateral tibial nerves. Improved SNAP was noted in the left sural nerve after treatment. Amp.: amplitude; Freq.: frequency; Lat.: latency; MNCS: motor nerve conduction study; MNCV: motor conduction velocity; SNCS: sensory nerve conduction study; SNCV: sensory conduction velocity *Improving the nerve conduction study of the left sural nerve.

MNCS	Pre-treatment			Post-treatment		
Nerve	Lat. (ms)	Amp. (mV)	MNCV (m/s)	Lat. (ms)	Amp. (mV)	MNCV (m/s)
Right median nerve (Wrist/Below elbow)	4.5/7.9	7.0/7.8	53	3.6/7.6	8.2/7.9	55
Left median nerve (Wrist/Below elbow)	3.9/7.7	7.9/7.7	55	3.5/7.3	8.9/8.6	57
Right ulnar nerve (Wrist/Below elbow)	2.9/7.7	12.0/10.1	52	2.7/7.5	14.7/13.1	56
Left ulnar nerve (Wrist/Below elbow)	2.9/7.8	16.5/15.9	55	2.6/7.4	15.4/13.3	55
Right peroneal nerve (Ankle/fibular head)	4.8/11.5	7.0/7.6	46	3.9/10.4	8.6/8.6	48
Left peroneal nerve (Ankle/fibular head)	4.0/11.8	7.0/6.3	44	3.7/10.3	7.1/6.6	48
Right tibial nerve (Ankle/popliteal fossa)	3.8/13.4	16.9/12.4	40	3.2/11.2	6.1/4.7	46
Left tibial nerve (Ankle/popliteal fossa)	3.9/15.4	20.3/15.2	40	3.3/11.2	4.8/4.1	47
SNCS	Pre-treatment			Post-treatment		
Nerve	Lat. (ms)	Amp. (mV)	SNCV (m/s)	Lat. (ms)	Amp. (mV)	SNCV (m/s)
Right median nerve	2.8	14	50	2.5	14	56
Left median nerve	2.4	13	58	2.4	6	58
Right ulnar nerve	2.8	4	49	2.7	18	51
Left ulnar nerve	2.7	6	52	3.0	16	46
Right sural nerve	absent	absent	absent	absent	absent	absent
Left sural nerve	absent	absent	absent	3.3*	15*	42*
F-wave	Pre-treatment			Post-treatment		
Nerve	Lat. (ms)			Lat. (ms)		
Right median nerve	26.5			25.0		
Left median nerve	26.8			23.8		
Right ulnar nerve	25.6			26.4		
Left ulnar nerve	25.9			26.0		
Right peroneal nerve	44.5			46.8		
Left peroneal nerve	45.2			45.6		
Right tibial nerve	50.0			46.4		
Left tibial nerve	49.2			46.0		
H-reflex	Pre-treatment			Post-treatment		
Nerve	Lat. (ms)	Amp. (mV)		Lat. (ms)	Amp. (mV)	
Right tibial nerve	absent	absent		28.8	0.2	
Left tibial nerve	absent	absent		29.1	0.3	

**Figure 1:**
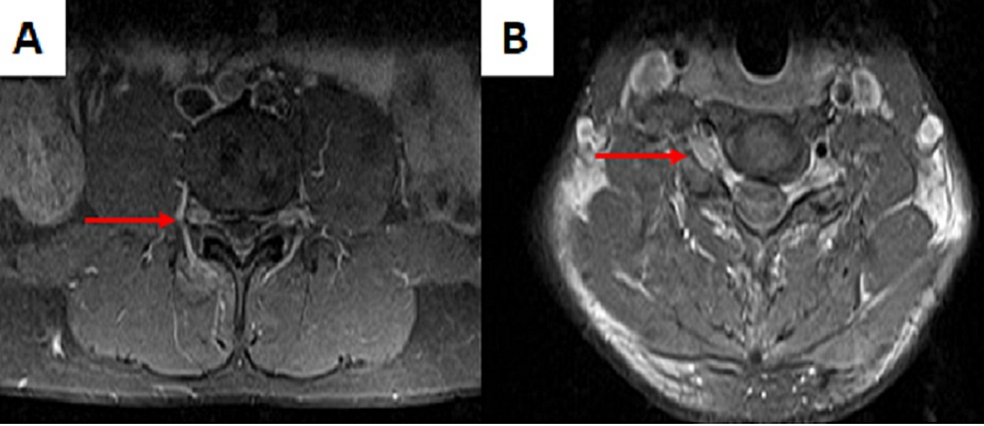
The MRI of the whole spine. Enlarged dorsal root ganglia (arrows) at the lumbar (A) and the cervical (B) regions (T1W gadolinium-enhanced, transverse view).

The somatosensory evoked potential (SSEP) study revealed prolonged interpeak intervals of EP-C7/C7-CTX following each median nerve stimulation and prolonged interpeak intervals of POP-L4 at the right tibial nerve. Blood tests revealed positive HLA-B27 without prominent increased erythrocyte sedimentation rate (ESR) or C-reactive protein (CRP) levels. The following serum tests were all within normal limits or negative: thyroid function, ß2 glycoprotein, vitamin B12, serum protein immunoelectrophoresis, rheumatologic antibodies, paraneoplastic antibodies, syphilis, and anti-HIV antibodies. The patient also had no symptoms suggestive of Sicca syndrome. Elevated protein levels without oligoclonal bands and normal immunoglobulin G (IgG) index were found in cerebrospinal fluid (CSF) studies. Enlarged DRG with post-gadolinium enhancement was seen on the whole spine magnetic resonance imaging (MRI) (Figures 1A, 1B).

**Figure 2:**
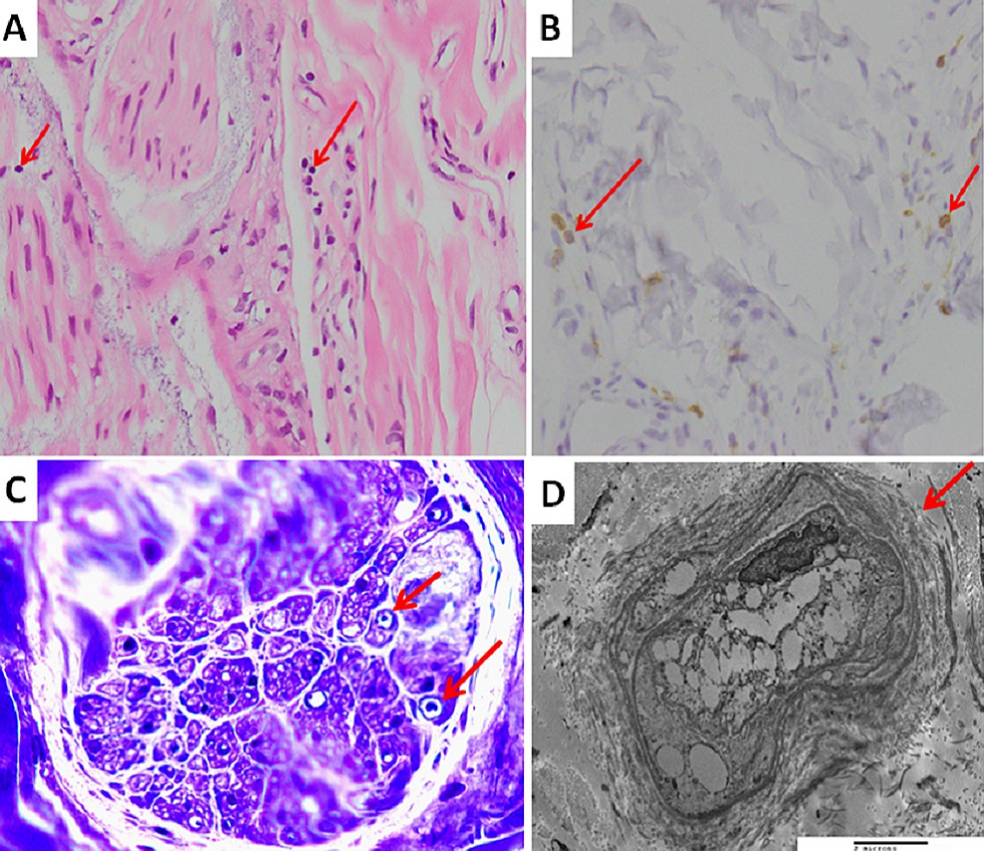
The pathologic findings of the left sural nerve biopsy. (A) Increased lymphocyte infiltration highlighted by CD45 immunostain (arrow), original magnification ×400. (B) Increased infiltration of lymphocytes in endoneurium (arrows), hematoxylin and eosin stain, original magnification ×400. (C) Decreased myelinated fibers in the epon semithin section (arrow). The nerve fibers were mostly unmyelinated, toluidine blue stain, original magnification ×1,000. (D) Electromicroscopic study: myelinated axon with a concentric arrangement of Schwann cell processes with onion bulb appearance is found (arrow), original magnification ×10,000.

The biopsy of the left sural nerve showed no amyloid deposition in Congo red stain, and also no evidence of vasculitis was found. Immunohistochemical stains for leukocyte common antigen (CD45), CD3, CD20, and CD68 were done and the results were consistent with inflammatory neuropathy with increased CD3+ T lymphocytes and CD68+ macrophages in the nerve tissue. The nerve bundles were mainly composed of unmyelinated axons in the electromicroscopic study and on epon semithin section stained with toluidine blue stain and degenerative axons were also seen. Myelinated axon with a concentric arrangement of Schwann cell processes with onion bulb appearance was rarely found (Figures 2A-2D).

The myelinated nerve fiber density was 223.52/mm^2^. The above-mentioned pathological findings were consistent with inflammatory demyelinating neuropathy.

We used the questionnaires with clinical total neuropathy score (TNSc), 36-item short-form health survey (SF-36), neuropathic pain symptom inventory (NPSI), and Pittsburgh sleep quality index (PSQI) to evaluate the treatment response. Only mild improvements were observed after pulse therapy with methylprednisolone 1,000mg/day for three days. The patient was then treated with human intravenous immunoglobulin (IVIg) with a dose of 2g/kg-BW, and the tingling sensation was reduced. Later we added azathioprine 25mg/day, and there was also an improvement in the tingling sensation in his right hand. After we titrated up the dose of azathioprine to 50mg/day, there was only residual tingling sensation at the fingertips and toes, and more muscle strength was reported. The loss of vibration and joint position sense subsided at bilateral ankles but still remained at the toes. According to the above-mentioned clinical features and treatment response to immune therapy, a diagnosis of CISP-plus was made.

Follow-up NCS after two, eight, 12, and 48 months still showed reduced SNAP amplitudes in bilateral sural nerves despite azathioprine use; however, gradual clinical improvements were noticed (Table 1).

No more tingling sensation was reported on the 12-month and 48-month clinic follow-up. The patient also received a spine MRI following after four years of disease onset, and the size of the previously enlarged DRG had decreased.

## Discussion

Chronic inflammatory demyelinating polyneuropathy (CIDP) can be classified into typical and atypical variants, and the latter includes Lewis-Sumner syndrome (LSS), distal acquired demyelinating symmetric (DADS) neuropathy, pure motor and pure sensory (including CISP) subtypes [5-8]. CIDP preferentially involves the nerves located proximal to the sensory ganglion cell, and the previous case reports mentioned much relative preservation of the sural nerve function in CISP [1,2,9]. CISP-plus is a newly introduced concept by Shelly et al., and it is different from CISP in that these patients had mild abnormalities on NCS that do not fully explain the clinical syndrome [3].

According to the literature review, to differential CISP from pure sensory CIDP, the abnormal SSEP findings could assist the diagnosis of CISP [7]. The sural nerve biopsy is also particularly supportive in the diagnosis of CISP and other atypical CIDP and could be even more sensitive than electrophysiology in diagnosing sensory neuropathies [10,11].

Other etiologies of sensory ataxia, such as spinocerebellar ataxia, sensory neuropathy of Sjogren syndrome, tabes dorsalis were excluded in the above-mentioned case after serial exams. In our case, the findings of hyporeflexia in four limbs, hypoesthesia by vibration, joint position and pinprick tests at bilateral palms and soles, sensory ataxia, cytoalbuminologic dissociation findings in CSF analysis, abnormal sensory NCS findings, proximal sensory dysfunction suggested by the SSEP which was not related to central nervous system involvements, enlarged DRG seen on the MRI, and the adequate response to immune-modulating therapy can support the diagnosis of CISP-plus.

Though sural nerve biopsies showed a broad spectrum of changes from no abnormalities, edema, demyelination, formation of onion bulbs, axonal degeneration, macrophage, and T-cell infiltrations from a literature review [6], it still could be considered for diagnostic assistance as the EFNS 2010 guideline suggested [12]. According to a study of morphometric analysis data of normal sural nerve, the normal range of myelinated fiber density of sural nerve was 3,585.5 to 10,872.3/mm^2^ and the average was 6,714.2 ± 1,560.7/mm^2^ [13]. Another literature mentioned of the mean myelinated fiber density of five CISP-plus patients was 5,724 ± 1,864/mm^2^ [3]. In our patient, the myelinated fiber density was markedly reduced compared to the normal range and also to other reported CISP-plus patients. The sural nerve was not involved in previous reported CISP cases, only one case had additional cranial nerve involvement, and another case showed median, ulnar and radial nerve involvements [9,14]. Even in the literature that first mentioned CISP-plus, the sural nerve NCS findings of all included CISP and CISP-plus patients were all within normal limits [3]. Nevertheless, in our patient, prominent sural nerve dysfunction was detected in the electrophysiological and pathological studies. The possible mechanism of sural nerve involvement in our patient has not been yet clarified, and there has been no other similar research reporting this. The patient was tested HLA-B27 positive; however, the diseases associated with HLA-B27 including psoriasis, ankylosing spondylitis, inflammatory bowel disease, and reactive arthritis were excluded. HLA associations are common in days of immune conditions. The role of HLA polymorphisms in influencing the clinical expression of CIDP has been studied and HLA-DR3 and DR3/DQ2 were significantly more frequent in CIDP patients; however, no relationship between HLA-B27 and CIDP was discovered [4]. Further investigations of the related immune mechanisms, associations between the HLAs and CIDP, and more detailed pathological studies may be required.

## Conclusions

The findings in our case suggesting sural nerve involvement could be possible in patients whose symptoms and results of electrophysiological studies had met the diagnostic criteria of CISP-plus. The pathologic reports of the biopsied sural nerve were also supportive and might be necessary for the diagnosis. Other than steroid or immunosuppressants, IVIg treatment is also effective for CISP-plus. Further studies are required to determine the possible immune mechanisms between CISP and the positive HLA-B27.
